# A new molecular mechanism underlying the EGCG-mediated autophagic modulation of AFP in HepG2 cells

**DOI:** 10.1038/cddis.2017.563

**Published:** 2017-11-02

**Authors:** Lin Zhao, Shengtang Liu, Jiaying Xu, Wei Li, Guangxin Duan, Haichao Wang, Huilin Yang, Zaixing Yang, Ruhong Zhou

**Affiliations:** 1 School of Radiological and Interdisciplinary Sciences (RAD-X), Collaborative Innovation Center of Radiation Medicine of Jiangsu Higher Education Institutions, Soochow University, Suzhou 215123, China; 2 The Feinstein Institute for Medical Research, 350 Community Drive, Manhasset, NY 11030, USA; 3 Department of Orthopedics, The First Affiliated Hospital of Soochow University, Soochow University, Suzhou 215006, China; 4 Computational Biological Center, IBM Thomas J Watson Research Center, Yorktown Heights, NY 10598, USA; 5 Department of Chemistry, Columbia University, New York, NY 10027, USA

## Abstract

Epigallocatechingallate (EGCG) is a major bioactive component of green tea and is associated with health benefits against multiple diseases including cancer. As an indicator of hepatocellular carcinoma (HCC), high levels of *α*-fetal protein (AFP) are related to malignant differentiation and poor prognosis of cancer cells. In this study, EGCG can effectively reduce AFP secretion and simultaneously induce AFP aggregation in human HCC HepG_2_ cells. EGCG-stimulated autophagy induces the degradation of AFP aggregates in HepG_2_ cells. Furthermore, we thoroughly studied the underlying molecular mechanisms behind EGCG-stimulated autophagy by using large-scale all-atom molecular dynamics simulations, which revealed a novel molecular mechanism. EGCG directly interacts with LC3-I protein, readily exposing the pivotal Gly-120 site of the latter to other important binding partners such as 1,2-distearoyl-*sn*-glycero-3-phosphoethanolamine and promoting the synthesis of LC3-II, a characteristic autophagosomal marker. Our results suggest that EGCG is critical in regulating AFP secretion and in modulating autophagic activities of HepG_2_ cells, providing a molecular basis for potentially preventing and treating HCC.

Autophagy is pivotal in various physiological processes. As a highly conserved degradation process of injured protein, lipid, and organelle, autophagy participates in cell growth, development, and death.^1^ Autophagy is also a necessary process in bone growth,^2^ while suppression of autophagy was related to pathologies, such as cancer.^3^ Traditionally, autophagosomal membrane formation is a key process during early autophagic stages. During this process, the microtubule-associated protein 1A/1B-light chain 3 (LC3-I) connects to 1,2-distearoyl-*sn*-glycero-3-phosphoethanolamine (DSPE) through an amide bond with the Gly-120 residue located in the C-terminal region of the former. Then, the formation of lipid-anchored protein LC3-II generates autophagosome production and promotes autophagic activity. When autophagosomes fuse with lysosomes, autolysosomes ultimately form, subjecting damaged organelles and protein aggregates to degradation.^4^ Recently, growing interest has focused on exploring the mechanisms of autophagy at the molecular level.

As a healthy beverage consumed worldwide, green tea is historically associated with enormous health benefits against multiple diseases including cancer.^5^ Numerous studies have intensively explored epigallocatechingallate (EGCG), which is the most abundant polyphenol in green tea, as a potential therapeutic agent because of its anti-inflammatory, antioxidant, antiobesity, and anticancer activities.^5, 6, 7^ In addition to advantages of oral administration and limited toxicity, recent studies have used nanoparticles as delivery vehicles to improve the low stability and bioavailability, further promoting the clinical application of EGCG.^8, 9^ In recent years, the roles of EGCG in mediating apoptotic or autophagy-induced cell death have received great attention, but the findings are controversial. On the one hand, EGCG promotes apoptosis and autophagy in oral cancer SSC-4 cells,^10^ and on the other hand, autophagy inhibition contributes to the synergy between EGCG and doxorubicin in a combined treatment of hepatoma Hep3B cells.^11^ A recent study also demonstrated that EGCG could attenuate apoptosis and autophagy in concanavalin A-induced hepatitis by inhibiting BNIP3.^12^ Therefore, the fact of whether EGCG indeed induces autophagy remains unclear. If EGCG does mediate autophagy, then we need to determine the molecular basis of its mechanism.

*α*-Fetal protein (AFP) is a major plasma protein produced by the yolk sac and the liver during fetal development, and represents the most abundant plasma protein (40–4000 *μ*g/ml) in the fetus. Although recent studies found that other circulating microRNAs, such as miR-122 and let-7b, performed some similar functions as AFP, AFP still serves as the most common marker in differentiating between hepatocellular carcinoma (HCC) and hepatic cirrhosis.^13^ As a prominent biomarker of HCC or germ cell tumors, high AFP level is connected to malignant differentiation, metastasis, and poor prognosis of cancer cells.^14^ According to recent statistics, HCC has become one of the leading cancers worldwide, and its mortality is increasing because of the lack of effective treatments against invasion and metastasis.^15^ Hence, exploration of potential AFP inhibitors is urgent to protect HCC patients from deterioration. Recently, Fang *et al.*^16^ showed that silencing of AFP expression by small interfering RNAs resulted in the effective inhibition of hepatoma cell growth and promotion of apoptosis. Houessinon *et al.*^17^ reported that sorafenib, a regular HCC chemotherapy drug, could control HCC, and its curative effect was best associated with lower AFP levels. In addition, a novel method combined anti-AFP-coated magnetic Fe_3_O_4_ nanoparticles with low-frequency electromagnetic field exposure induced the apoptosis of Bel-7402 and HepG_2_ hepatoma cells lines without manifesting any significant side effects on HL-7702, a normal hepatic cell line.^18^ Notably, EGCG could inhibit AFP secretion in human hepatoma-derived PLC/PRF/5 cells^19^ and reduces the serum AFP level in HCC rat models.^20^ However, the mechanism underlying the way by which EGCG regulates AFP levels in HCC cells remains elusive. By using a combined approach with both experimental and computational techniques, we aim to demonstrate the effect of EGCG on AFP secretion, and more importantly, to reveal the underlying relationship between AFP secretion and EGCG-induced autophagic activities in human HCC HepG_2_ cells.

## Results

### EGCG inhibits the growth of HepG2 cells

**Figure 1:**
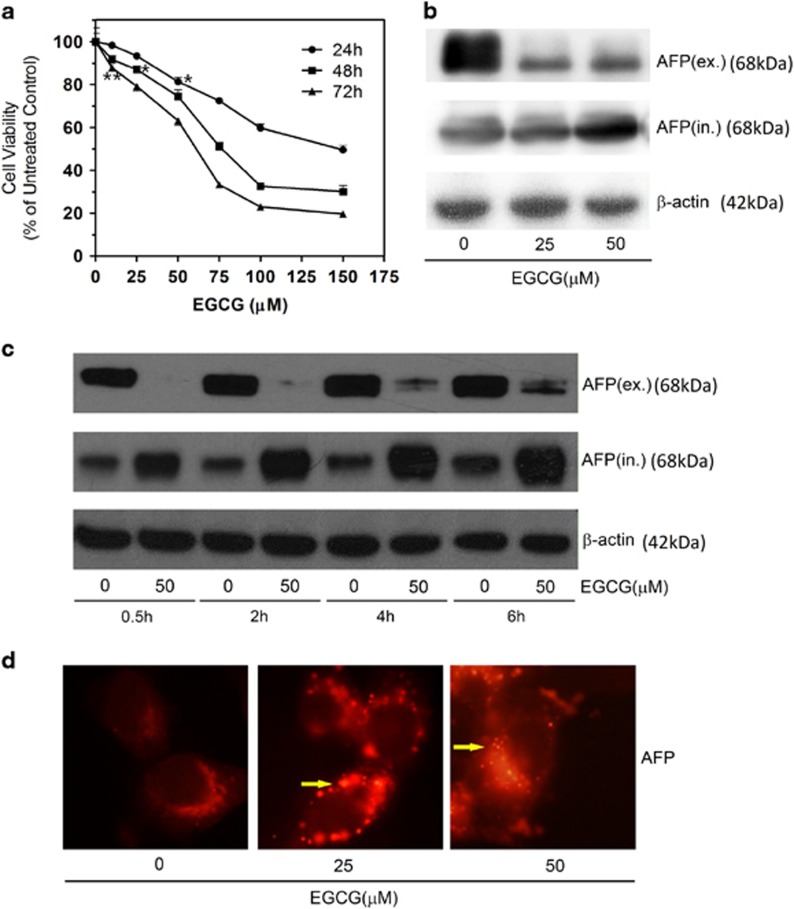
EGCG induces AFP aggregation in HepG_2_ cells. (**a**) MTT assay of HepG_2_ cells after 0–150 *μ*M EGCG treatment (with FBS) for 24, 48, and 72 h. *P<0.05 (*t*=2.72 for 24 h, *t*=2.83 for 48 h) and **P<0.01 (*t*=5.30 for 72 h), the treated groups were compared with an untreated control (considered to be 100% viable). The data are presented as mean±S.D. derived from three independent experiments. (**b**) Western blot assay of intracellular and extracellular AFP. HepG_2_ cells were treated in the absence or presence of 25 and 50 *μ*M EGCG for 24 h. (**c**) HepG_2_ cells were incubated with or without 50 *μ*M EGCG for 0–6 h, and intracellular and extracellular protein were subjected for western blotting analysis. (**d**) HepG_2_ cells were treated with 0, 25, and 50 *μ*M EGCG for 24 h, respectively, and stained with AFP-specific primary antibody and CY3-conjugated secondary antibody. Yellow arrows: aggregated AFP protein (× 40)

We used the 3-[4,5-dimethyl-2-thiazolyl]-2,5-diphenyl-2*H*-tetrazolium bromide (MTT) method in our study to analyze the cytotoxic effect of EGCG on HepG_2_ cells. EGCG inhibited the growth of HepG_2_ cells in a time- and dose-dependent manner with IC_50_ values between 60 and 80 *μ*M (Figure 1a). At 150 *μ*M, the maximum inhibition of cell growth by EGCG was 50.44%, 69.96%, and 80.38% after 24, 48, and 72 h, respectively.

### EGCG reduced AFP secretion in HepG_2_ cells

We measured intracellular and extracellular AFP content by western blot analysis in HepG_2_ cells to further determine the influence of EGCG on AFP secretion. At concentrations below the 50 *μ*M threshold, EGCG significantly reduced the extracellular AFP level in a dose-dependent trend after 24 h treatment (Figure 1b). To a less extent, the elevation of intracellular AFP levels in HepG_2_ cells depended on EGCG dose during incubation (Figure 1b). EGCG-mediated inhibition of AFP secretion was also time-dependent, occurring within 0.5 h, but lasting up to 6–24 h (Figure 1c). Immunocytochemistry analysis revealed that EGCG treatment resulted in cytoplasmic AFP aggregates manifesting as numerous 'puncta' structures in HepG2 cells (Figure 1d). Taken together, these experimental data suggested that EGCG treatment caused cytoplasmic AFP aggregation, and inhibited AFP secretion in HepG_2_ cells.

### EGCG induces mitochondria and cytoskeleton damage

To gain insight into the mechanism of EGCG-mediated inhibition of AFP secretion, we examined mitochondrial membrane potential and cytoskeleton structure by fluorescence-labeling. In normal healthy cells, the JC-1 molecular probe permeated into the mitochondrial matrix to form JC-1 aggregates that produced red fluorescence. When the mitochondrial potential decreased, such as in the case of apoptosis onset, the JC-1 molecular probe could not enter the mitochondria and emitted a green fluorescence instead. In the presence of 25 and 50 *μ*M EGCG, only short (0.5 h) exposure could result in a rapid decrease of red fluorescence, which became further pronounced as incubation time progresses to 2 and 4 h, indicating that EGCG stimulated the quick decrease of mitochondrial potential (Supplementary Figure S2A). In comparison with the untreated control where the actins in the green-labeled cytoskeleton were observably continuous, some cytoskeleton structures appeared cloddy after treatment with 25 and 50 *μ*M EGCG for 4 h, indicating the interruption of normal actin structure and precipitation of dysregulated actin aggregate by EGCG (Supplementary Figure S2B)

### EGCG promoted LC3-II production and autophagic AFP degradation

**Figure 2:**
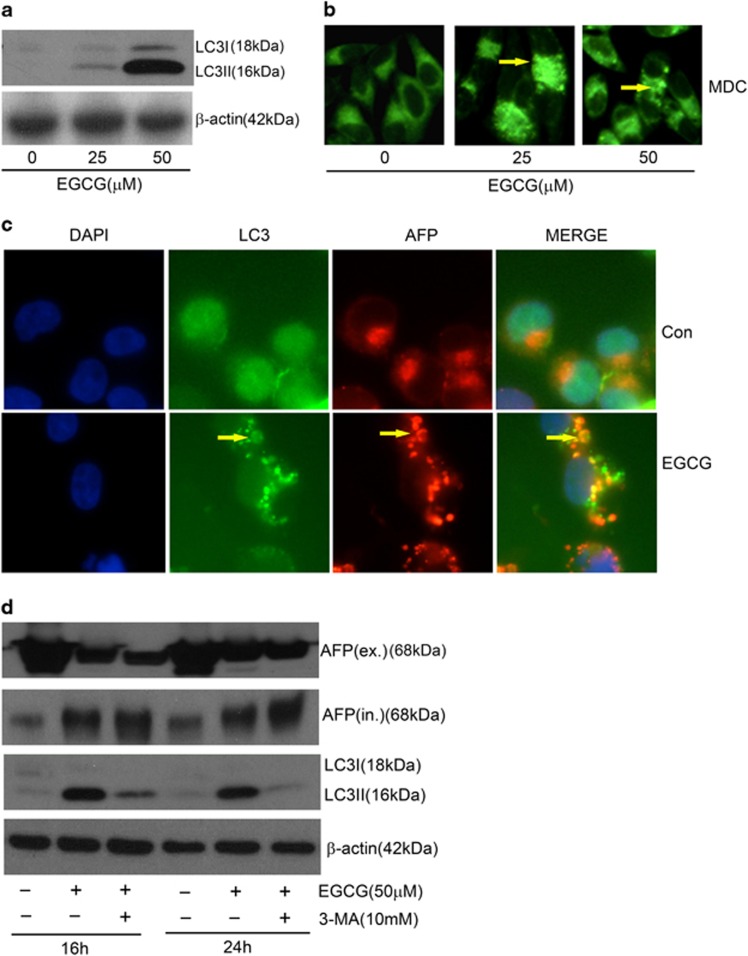
EGCG induced intracellular AFP degradation through autophagy in HepG_2_ cells. (**a**) HepG_2_ cells were stimulated with 25 and 50 *μ*M EGCG for 24 h. The 18-kDa cytosolic LC3-I and 16-kDa lipidated autophagosome-bound LC3-II was analyzed by the western blot method. (**b**) HepG_2_ cells were stimulated with 25 and 50 *μ*M EGCG for 24 h, and then stained with acidotropic MDC dye to visualize autophagosomes (yellow arrow: autophagosomes) (× 40). (**c**) GFP-transfected HepG_2_ cells were stimulated with 50 *μ*M EGCG for 24 h. Cytoplasmic aggregated AFP were partly colocalized with LC3-containing vesicles (as shown by yellow arrows) (× 60). (**d**) EGCG enhanced intracellular AFP level in the presence of 3-MA. HepG_2_ cells were stimulated with 50 *μ*M EGCG for 16 and 24 h, and 10 mM 3-MA was added at 4 h before EGCG treatment. Western blot analysis was conducted to determine extracellular, intracellular LC3, and AFP level with reference to *β*-actin

Consistent with a previous report,^21^ EGCG elevated LC3-II production in non-transfected HepG_2_ cells (Figure 2a). Moreover, EGCG induced the formation of LC3-containing cytoplasmic vesicles in GFP-LC3-transfected HepG_2_ cells (autophagosomes, Figure 2c, yellow arrow). Similarly, monodansylcadaverine (MDC) nonspecific stain had also shown the autophagic vacuoles (Figure 2b, yellow arrow). Thus, EGCG could increase LC3-II production and stimulate autophagy. To further test the possibility of autophagic degradation gradually eliminating the AFP aggregates, we determined the colocalization of AFP aggregates in autophagosomes. As we predicted, many cytoplasmic AFP 'puncta' appeared colocalizing with LC3-containing cytoplasmic vesicles after 24 h treatment with 25 *μ*M EGCG (Figure 2c). Furthermore, the blockage of autophagy with 3-methyladenine (3-MA), a specific inhibitor of autophagy-regulating molecule, could further promote the accumulation of cytoplasmic AFP, simultaneously reducing LC3-I and LC3-II levels (Figure 2d). These results collectively suggest that EGCG-mediated AFP aggregation may facilitate autophagic degradation in HepG2 cells.

### Spontaneous dimerization of LC3-I in pure water impedes formation of LC3-II complex

**Figure 3:**
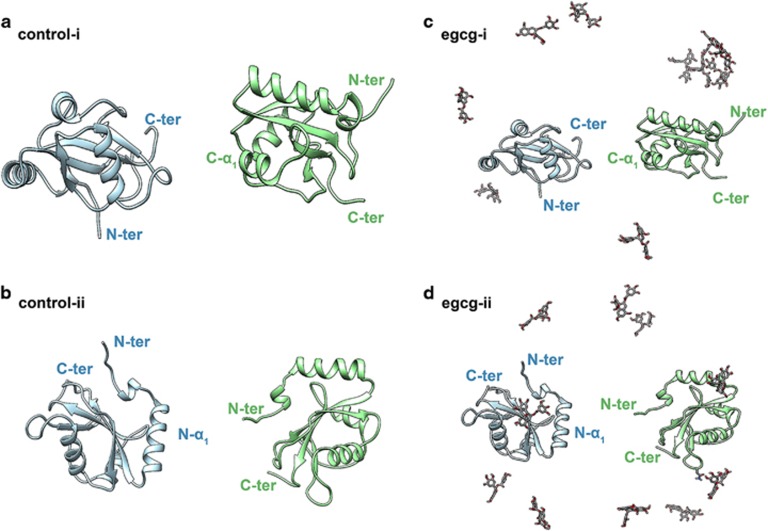
The initial configurations for molecular dynamic simulations consisting of LC3-I protein and EGCG molecular in the water solvated system. LC3-I protein inserted in pure water marked as the control system: (**a**) control-i, in the C-terminal region of two LC3-I proteins faced each other; (**b**) control-ii system, in the N-terminal region of two LC3-I proteins faced each other. Ten EGCG molecules were randomly placed into (**a**) and (**b**) systems, yielding two corresponding EGCG+ system: (**c**) EGCG-i and (**d**) EGCG-ii, respectively. For clarity, water molecules and ions are not shown. Two LC3-I protein are shown as light green and light blue NewCartoon representation. EGCG molecules colored by element, carbon atoms are gray, oxygen atoms are red, and hydrogen atoms are white

**Figure 4:**
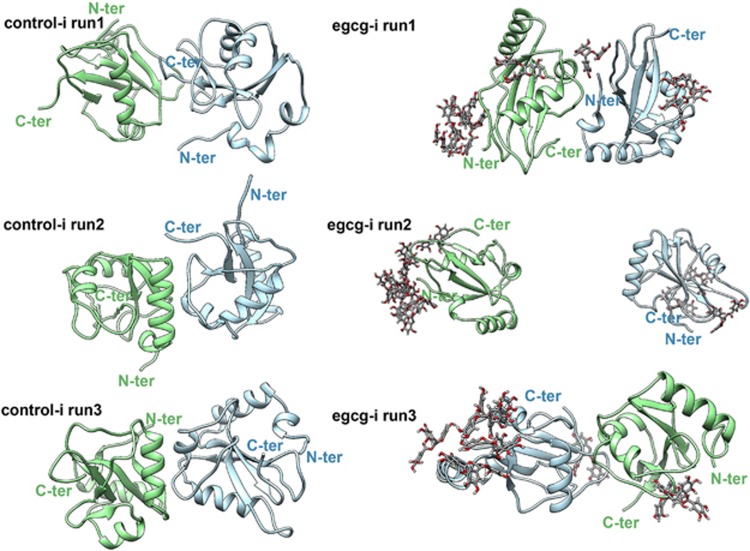
The three final snapshots (at *t*=100 ns) of control-i (left column) and EGCG-i system (right column, system depicted in Figure 3) from different initial phase space. LC3-I proteins are displayed with different color to distinguish them, and the terminal of proteins were labeled as N-ter and C-ter for the same purpose

The effect of EGCG on the dimerization of LC3-I proteins and its potential influence on the formation of LC3-II complex were furtherly explored with all-atom molecular dynamic (MD) simulations. In control simulations, the two individual LC3-I proteins (independent of the initial orientation, Figures 3a and b) aggregated to a stable and morphologically similar dimer in all trajectories (Figure 4, left column). Time-based evolution of the distance between the center of mass (COM) of two proteins reveals the quick nature of the dimerization process (Supplementary Figure S3). The dimerization patterns for all control simulations are very similar, that is, the N-*β*_2_ and N-*α*_2_ regions in the N-terminal region of a LC3-I protein bind to the C-*β*_1_ region in the C-terminal region of another LC3-I protein (Figure 4 and Supplementary Figure S4). The formed dimers in pure water were structurally closed, with at least one Gly-120 site on the interface of two LC3-I proteins completely harbored and inaccessible for DSPE. Numerous studies have fully addressed the role of Gly-120 in the formation of LC3-II complex,^22, 23, 24^ with the conjugation of Gly-120 site of LC3-I protein to DSPE through an amide bond being the most important step.^22^ Therefore, in pure water, dimerization of LC3-I proteins is unfavorable for the formation of LC3-II complex.

### EGCG promoted LC3-II production by inhibiting dimerization of LC3-I

After adding EGCG, the dimerization of LC3-I undergoes a noticeable delay or somewhat inhibition. For example, in some EGCG+ simulations, the two dimers remaining separated from each other till the end of the simulations (run2 in EGCG-i and EGCG-ii system, the middle picture in right column of Figure 4 and Supplementary Figure S3, respectively). The much larger averaged COM distance between two monomers in EGCG-ii systems (~3.57±0.49 nm) in comparison with control systems (~2.96±0.50 nm) also demonstrated the inhibition of LC3-I protein dimerization (Supplementary Figure S3). In run1 of the EGCG-i system (the purple curve in Supplementary Figure S3), the COM distance between two monomers largely fluctuates until *t*>70 ns, when the two monomers begin to approach each other. In run2 (the green curve in Supplementary Figure S3, EGCG-i system), the two monomers did not even show any dimerizing tendency. Taken together, the dimerization of LC3-I was significantly inhibited. Consequently, the Gly-120 site in the C-terminal region of LC3-I protein is freely open to DSPE (the right column of Figure 4 and Supplementary Figure S4), which in turn can promote the formation of the LC3-II complex and result in the degradation of AFP aggregates by autophagy.

### The underlying molecular mechanisms for EGCG inhibited dimerization of LC3-I

**Figure 5:**
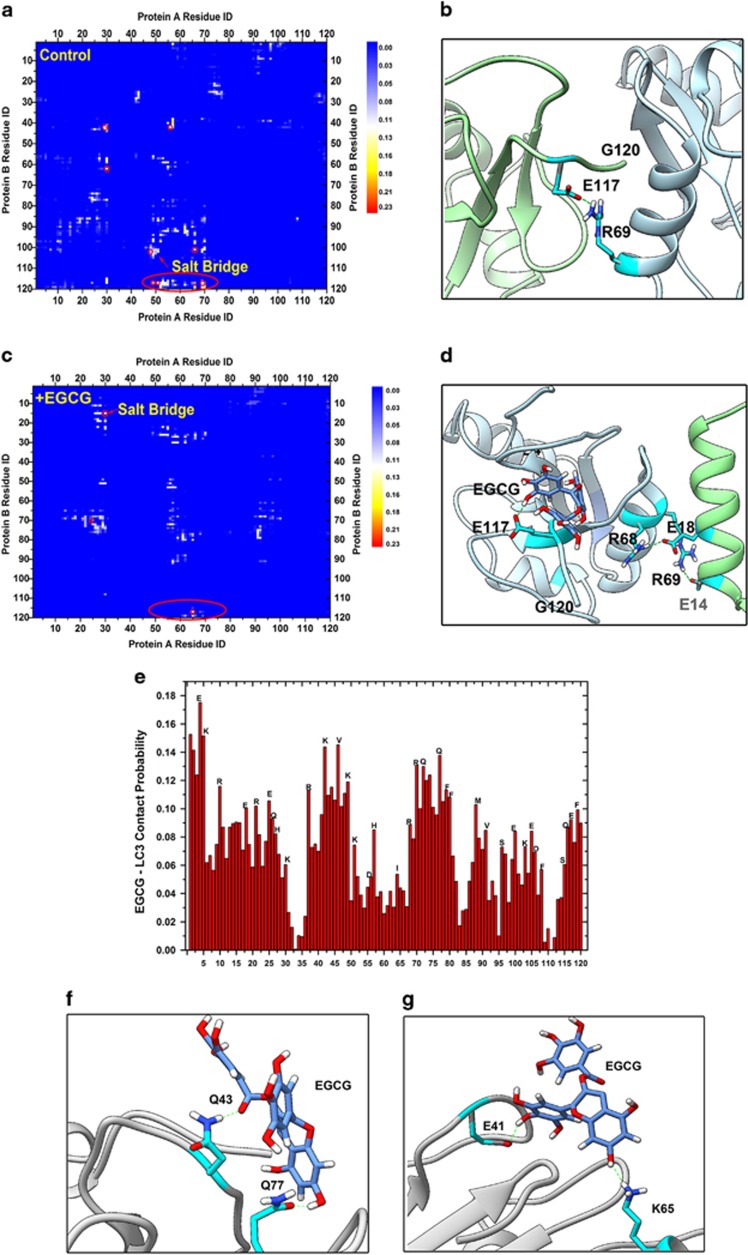
The underlying molecular mechanisms for EGCG to inhibit the dimerization of LC3-I protein. The residual contact probability map of two LC3-I protein (**a**) is counted over all control systems, and (**c**) is counted over all the EGCG+ systems. The contact cutoff is defined as 0.5 nm. (**b** and **d**) The corresponding typical configurations highlighted by the red circle in (**a**) and (**b**), respectively. (**e**) The contact probability of EGCG with LC3-I protein (cutoff is 0.5 nm). The typical configurations for EGCG interacting with the polar residues (**f**), and the charged residues (**g**) through hydrogen bonds

Furthermore, to gain a deeper insight into molecular mechanisms of EGCG-stimulated autophagosome formation, namely, the interaction between EGCG with LC3-I protein, the residual contact probability between two LC3-I monomers were analyzed. The formation of many stable salt-bridges between two proteins, particularly Glu-117 with Lys-48, Lys-50, Lys-65 or Arg-69, Arg-70, mainly triggered LC3-I dimerization (Figure 5a). Figure 5b shows a typical LC3-I protein dimer stabilized by a salt-bridge between Glu-117 and Arg-69, which directly induces the complete harboring of the Gly-120 site on the interface of two LC3-I proteins, thus preventing access of DSPE, and hindering the formation of LC3-II complex. Upon addition of EGCG, the residual contact probability map between two proteins further illustrates the suppression of salt-bridge formation during protein dimerization (Figure 5a), particularly on the residues around Glu-117 (Figures 5c and d). Thus, the Gly-120 site in the C-terminal region of LC3-I protein is released to DSPE. The capability of EGCG inhibiting LC3-I dimerization mainly arises from the high binding affinity of the former with the charged and polar residues of the latter through electrostatic attractions and hydrogen bonding, respectively (Figures 5e–g). The coating of EGCGs (especially the charged sites) acts as a shield against long-range electrostatic interactions between two proteins and inhibits the dimerization of proteins.

## Discussion

Accumulating scientific reports have extensively studied EGCG, the most active and abundant component of green tea, for its anticancer effect. Previously, the effect of the apoptotic mechanism stimulated by lower mitochondrial membrane potential and the G0/G1 phase cell cycle arrest was previously demonstrated in HCC.^25^ In addition, other reports have indicated that targeting the EP(1) receptor^26^ and copper accumulation^27^ can mediate anti-HCC activities. In our study, EGCG at concentrations below 50 *μ*M could inhibit HepG_2_ cell growth slightly better than higher EGCG concentrations of 50–100 *μ*M under the same conditions (Figure 1a). Thus, we proposed that EGCG concentrations below 50 *μ*M should be capable of evoking protective mechanisms that battle against its damaging effect.

**Figure 6:**
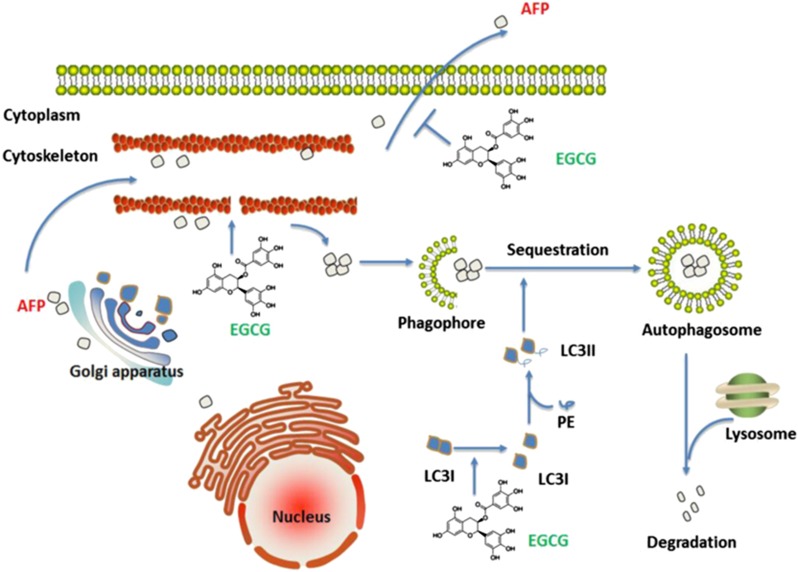
The mechanism of EGCG inhibits AFP secretion and promotes its degradation by autophagy

In the present study, EGCG diminished the secretion of AFP (Figures 1b and c). AFP seemed trapped in the cytoplasm of HepG_2_ cells after EGCG treatment (Figure 1d). As a universal biomarker of HCC, AFP is generally detectable in patients with hepatological diseases. We hypothesized that EGCG possibly inhibited AFP secretion by causing an energy shortage in the mitochondria and a deficiency of motion-dependent matrix in the cytoskeleton (Supplementary Figure S2 and Figure 6). Intriguingly, EGCG lowered the level of secreted osteopontin, a protein similar to AFP, which also mediated HCC metastasis and invasion.^28^ Because a previous report demonstrated that secreted AFP could enhance tumor cell proliferation by binding to AFP receptors localized in HCC cell membranes,^29^ we considered that the diminished AFP secretion by EGCG should be one of the reasons behind the inhibition of HepG_2_ growth.

As salvation of dying cells, autophagy participates in numerous cellular events. According to previous studies, the possibility of EGCG promoting or attenuating autophagy remains elusive. Recent studies had indicated that EGCG exhibited an antiautophagic effect through the IL-6/JAKs/STAT3 pathway,^12^ or protection of mitochondrial function,^30^ benefitting the organism. Paradoxically, EGCG could potentially activate autophagy in certain cases to protect normal cells^31^ or sensitize the cancer cells to chemotherapy drugs.^32^ EGCG could notably increase autophagosome synthesis instead of inhibiting LC3-II degradation upon adding a proton pump inhibitor, such as bafilomycin A1, at saturating concentrations in raw cells.^21^ Our results supported this opinion, and we proposed that autophagy should be one way of degrading intracellular AFP (Figure 6). Several previous studies concluded that cytoplasmic AFP could activate the (PI3K)/AKT pathway,^33^ prevent RAR from nuclear translocation, and inhibit the expression of *Fn1*4 gene,^34^ which participates in the regulation of HCC growth. Thus, autophagy is important in complementing the protective effect of EGCG and in fighting against damage.

Our MD simulation further explored the underlying mechanism of EGCG-promoted LC3-II production. It was suggested that EGCG binding to the charged residues or other polar residues of LC3-I protein can effectively hinder the long-range electrostatic interactions between two monomer proteins, and thereby inhibit the dimerization of LC3-I. Inhibition of the dimerization of proteins leads the pivotal Gly-120 site in the C-terminal region of LC3-I more exposed to the other molecules such as DSPE, which in turn can evaluate the production of LC3-II complex (Figure 6). Historically, there are numerous studies discussing about the interaction of EGCG with proteins. It was illustrated that EGCG possessed the therapeutic potential against Alzheimer's disease, and had been recognized as an antiamyloid agent.^35, 36^ In addition, EGCG inhibited the aggregation of tau into toxic oligomers, and protected the neuronal model cells.^37^ Furthermore, we also found that EGCG could firmly bind to HMGB1 near Cys-106, and subsequently stimulated large HMGB1 conformational change.^38^ All these indicted that EGCG possessed unique functions, which determined its diverse applications in biomedical field.

In conclusion, we deduced the complex biochemistry and physical reactions in human HCC HepG_2_ cells treated with EGCG at concentrations below 50 *μ*M. On the one hand, EGCG displayed its cytotoxic effects by inhibiting AFP secretion and promoting intracellular aggregation. On the other hand, EGCG also evoked autophagy to degrade the AFP aggregate and to protect cells from damage. Furthermore, our findings provide a novel mechanism on the autophagic function of EGCG towards AFP and the unique advantages of autophagy in the prevention and treatment of liver cancer, supporting the health benefits of green tea.

## Materials and methods

### Reagents

EGCG (≥98% purity), 3-MA and 4′,6-diamidino-2-phenylindole (DAPI), MDC, and MTT were provided by Sigma (St. Louis, MO, USA). EGCG was dissolved in deionized water to prepare 10 mM stock solutions at −20 °C. Dulbecco’s modified Eagle’s medium (DMEM), OPTI-MEM I medium and fetal bovine serum (FBS) were purchased from Gibco (Grand Island, NY, USA). AFP and LC3 mouse monoclonal antibody were purchased from Santa Cruz Biotechnology (Santa Cruz, CA, USA). Anti-*β*-actin was obtained from Sigma-Aldrich (St. Louis, MO, USA). Horseradish peroxidase-conjugated anti-rabbit or anti-mouse secondary antibodies were purchased from Wuhan Boster Biological Engineering Co. (Wuhan, China). Cy3-labeled anti-mouse antibody, Actin-Tracker Green, Mitochondrial Membrane Potential Assay Kit with JC-1 and the BCA Protein Assay Kit were purchased from Beyotime Institute of Biotechnology (Beijing, China).

### Cell culture

HepG_2_ cells were obtained from the American Type Culture Collection (Rockville, MD, USA). Cells were maintained in DMEM (Gibco, Shanghai, China), supplemented with 10% fetal bovine serum (FBS), l-glutamine (5 mmol/l), penicillin (100 U/ml), and streptomycin (100 U/ml) (Invitrogen, Carlsbad, CA, USA), at 37 °C in a humidified 5% CO_2_ atmosphere.

### Transfection and establishment of stable cell lines

HepG_2_ cells were transfected with GFP-LC3 plasmid (obtained from Dr. N Tony Eissa, Department of Medicine, Baylor College of Medicine, Houston, TX, USA) by Lipofectamine 2000 (Life Technologies Inc., Carlsbad, CA, USA), with an aim to detect specific autophagosome by using LC3-transfected HepG_2_ cells, which could help reflect the level of autophagy. Briefly, the Lipofectamine reagent (1 : 25, v/v) and the GFP-LC3 plasmid (1.0 *μ*g/1000 *μ*l) were diluted by the serum-reduced OPTI-MEM I medium in separate Eppendorf tube. Then, they were mixed at a 1 : 1 ratio (v/v), and then incubated at room temperature for 10 min. HepG_2_ cells (1–2 × 10^6^ cells) were seeded onto 100-mm tissue culture Petri dish (10 ml) and cultured in the DMEM/10% FBS/2 mM glutamine until reaching 70–80% confluency, and replaced with fresh OPTI-MEM I medium (10 ml) after two washes with prewarmed OPTI-MEM I medium. The GFP-LC3 plasmid/Lipofectamine mixture (5.0 ml, containing 2.5 *μ*g plasmid DNA) was added to the HepG_2_ cell culture, and incubated for 12–16 h. Afterwards, HepG_2_ cells stably expressing GFP-LC3 were selected and maintained in DMEM/10%FBS/2 mM glutamine supplemented with geneticin (600 *μ*g/ml; Gibco, China).

### Cell viability assay

MTT assay was performed to measure cell viability. Generally, HepG_2_ cells were seeded into 96-well cell culture plates at the density of 6000 cells/well. EGCG at different concentrations (0–150 *μ*M) were added to cells for 24, 48, and 72 h incubation. Then, the treated cells were incubated with 20 *μ*l MTT (5 mg/ml) in each well for another 4 h. The supernatant was removed and 150 *μ*l DMSO was added into each well, and after oscillating for a 10 min duration, the absorbance (OD value) was determined by a Micro Plate Reader (Bio-Tek Instruments Inc., Winooski, VT, USA) at 570 nm, with the absorbance at 630 nm as the background correction. The effect on cell proliferation was expressed as the percent cell viability. Untreated cells were taken as 100% viable.

### Mitochondrial membrane potential assay

JC-1 fluorescence probe was used to test the mitochondrial membrane potential. HepG_2_ cells were seeded into 6-well cell culture plates and incubated with 0, 25, and 50 *μ*M EGCG for 0.5, 2, and 4 h, respectively, when cells were attached onto the bottom of the plates. Then, after extensively washing with sterile PBS, the prepared 1 ml JC-1 working buffer mixed with 1 ml cell culture medium (containing FBS) were added into each well at 37 °C for 20 min. JC-1 dyeing buffer was discarded and the cells were washed with ice-cold PBS for two times to avoid the cytotoxic effect of JC-1. Later, the fresh medium was readded and images were captured using a fluorescence microscope (Carl Zeiss Microimaging Inc., Göttingen, Germany).

### Visualization of cytoskeleton

HepG_2_ cells were plated onto circular glass coverslips and incubated with different concentrations of EGCG (0, 25, and 50 *μ*M) for 6 h. Afterwards, cells were washed with PBS and fixed with freshly prepared 3.7% methanal for 10 min at room temperature, and permeabilized with 0.1% Triton X-100 in PBS three times for the next 5 min. Diluted Actin-Tracker Green (1 : 100) was added and incubated for 30 min at 37 °C. Then, the green actin cells inside was captured by a fluorescence microscope (Carl Zeiss Microimaging).

### Visualization of autophagosomes

HepG_2_ cells stably transfected with GFP-LC3 were treated in the absence or presence of EGCG for 24 h. And, the presence of GFP-LC3 punctate structures in cells was examined under a fluorescence microscope. In addition, MDC dyeing method was also used to detect unspecific autophagosomes. First, MDC was dissolved in DMSO to prepare 50 mM stock solution and diluted at 1:1000 for working solution. HepG_2_ cells were treated with 0, 25, and 50 *μ*M EGCG for 24 h, respectively. Then, cells were washed with PBS and dyed with MDC working solution for 30 min at room temperature. The images were captured by a fluorescence microscope (Carl Zeiss Microimaging).

### Immunofluorescence analysis

To visualize AFP localization, HepG_2_ cells were plated onto circular glass coverslips and incubated in the presence or absence of EGCG (25 and 50 *μ*M) for 24 h. To detect the colocalization of AFP and autophagosome, GFP-LC3-labeled HepG_2_ cells was treated with 50 *μ*M EGCG for 24 h. The immunofluorescence analysis was conducted as described previously.^39^ Briefly, cells were fixed with freshly prepared 2% formalin for 20 min at room temperature, and were permeabilized with 0.1% Triton X-100 in PBS for next 10 min. AFP antibody was added at 1 : 1000 and incubated overnight at 4 °C, followed by a mouse IgG-CY3 secondary antibody (Beyotime, Jiangsu, China) at 1 : 1000 for 1 h. The nucleus was stained with 5 *μ*g/ml DAPI at room temperature for 30 min. Images were captured using a fluorescence microscope (Carl Zeiss Microimaging).

### Western blotting analysis

HepG_2_ cells were incubated with or without 25 and 50 *μ*M EGCG for 24 h. To test the secreted AFP content in the medium, the culture supernatant was concentrated by an ultrafiltration device (Amicin Ultra-4 Centrifugal Filter Units; Millipore Co., Billerica, MA, USA), and then collected to conduct western blot analysis. Cellular total protein was extracted from cells using an IP lysing buffer (Beyotime, Jiangsu, China) containing (25 mg/ml) protease inhibitor (complete ULTRA Tablets; Roche, Mannheim, Germany). Protein concentration was determined by the BCA Protein Assay Kit (Beyotime, Jiangsu, China). The antibodies of AFP and LC3 were diluted at concentration 1 : 1000, and the western blot analysis was conducted as described previously.^39^ In addition, for testing the autophagic flux, we evaluated the LC3-II turnover after the treatment of EGCG in the absence or presence of an autophagy inhibitor, 3-MA. 10 mM 3-MA was added at 4 h before HepG_2_ cells were stimulated with 50 *μ*M EGCG for 16, 24 h, respectively. Cells were harvested and assayed for the ratio between the 18-kDa cytosolic LC3-I and 16-kDa lipidated autophagosme-bound LC3-II.

### Molecular dynamics simulations

The crystal structure of LC3-I protein was obtained from the Protein Data Bank (PDB code: 1V49^40^). Analyses of electrostatic surface potential (ESP) demonstrate that the LC3-I protein has large molecular polarity and distinctive electrostatic potential surface, with the positive charged region contributed by the N-terminal region, and the negative charged region contributed by the C-terminal region (see Supplementary Figure S1) (see below). Therefore, to avoid the quick approaching of two LC3-I proteins induced by the polarity of protein, and enrich sampling, the initial configurations of control systems were set to the C-terminal region (control-i system) or the N-terminal region (control-ii system) of two LC3-I proteins facing each other (see below). Initially, for both systems, the closest distances between any heavy atoms of two proteins were settled larger than 1.0 nm. Then, both protein dimers were solvated into a rectangular water box with a size of (13.64 nm × 10.51 nm × 13.32nm) and (13.13 nm × 11.39 nm × 9.56 nm) for control-i and control-ii systems (see below), respectively. Two Cl^−^ ions were added to neutralize both systems. After an equilibrium procedure (more below), 10 EGCG molecules were randomly introduced into the simulation boxes of control-i and control-ii systems, yielding two new EGCG+ systems (denoted by EGCG-i and EGCG-ii systems (see below)). These two EGCG+ systems were used to study the effect of EGCG on the dimerization of the LC3-I protein.

The molecular dynamic (MD) simulations were conducted with the software package GROMACS version 4.6.7.^41^ The protein ESP was depicted with APBS server.^42^ Chimera program^43^ was used for trajectory visualization and analysis. The GROMOS96 53a6 force field^44^ was used for proteins. The force field parameters for EGCG were adopted from a previous literature.^45^ The simple point charge model was used for water.^46^ The two control systems were subjected to a 20 000 steps energy minimization, followed by a further 2 ns NVT relaxation, with the temperature maintained at 300 K using *v*-rescale thermostat.^47^ The resulting two EGCG+ systems were re-equilibrated with the similar equilibration protocol as the two control systems. Then, for each system, 3 × 100 ns independent production runs were performed. The total aggregated simulation time was larger than 1.2 *μ*s. During the production runs, an NPT ensemble was used, with the temperature maintained at 300 K using v-rescale thermostat, and the pressure at 1 bar via Parrinello–Rahman coupling scheme.^48^ A time step of 2.0 fs was used, and data were collected every 5 ps. For all of our simulations, periodic boundary conditions were applied in all directions. The long-range electrostatic interactions were treated with the PME method,^49^ and the van der Waals interactions were calculated with a cutoff distance of 1.0 nm. All solute bond lengths were maintained constant at their equilibrium values with the LINCS algorithm,^50^ and water geometry was also constrained using the SETTLE algorithm.^51^

### Statistical analysis

Data are expressed as mean±S.D. of at least three independent experiments (*n*=3+). Statistical comparisons of the experimental results between the treated group and the control group were made using the two-tailed Student’s *t*-test. All statistical tests were performed using SPSS version 17.0 (IBM, Chicago, IL,USA). *P-*value ≤0.05 between groups was considered significant.

## Publisher’s Note

Springer Nature remains neutral with regard to jurisdictional claims in published maps and institutional affiliations.
