# Supplementary material for: A new molecular mechanism underlying the EGCG-mediated autophagic modulation of AFP in HepG2 cells
Source: Cell Death Dis. 2017 Nov 2;8(11):e3160–. doi: 10.1038/cddis.2017.563 (PMC5775413; doi:10.1038/cddis.2017.563)
Supplement: Supplementary Material [file cddis2017563x1.docx]

**Supporting Information**

**A new molecular mechanism underlying the EGCG-mediated autophagic modulation of AFP in HepG2 cells**

Lin Zhao^1,‡^, Shengtang Liu^1,‡^, Jiaying Xu^1^, Wei Li^4^, Guangxin Duan^1^, Haichao Wang^4^,Huilin Yang^5^, Zaixing Yang^1, *^, and Ruhong Zhou^1,2,3,*^

1. School for Radiological and Interdisciplinary Sciences (RAD-X) and Collaborative Innovation Center of Radiation Medicine of Jiangsu Higher Education Institutions, Soochow University, Suzhou, China, 215123

2. Computational Biological Center, IBM Thomas J. Watson Research Center, Yorktown Heights, NY 10598, USA

3. Department of Chemistry, Columbia University, New York, NY 10027, USA

4. The Feinstein Institute for Medical Research, 350 Community Drive, Manhasset, NY, 11030, USA.

5.Department of Orthopedics, The First Affiliated Hospital of Soochow University, Soochow University, Suzhou, China, 215006

E-mail: [ruhongz@us.ibm.com](mailto:ruhongz@us.ibm.com); [zxyang@suda.edu.cn](mailto:zxyang@suda.edu.cn)

**Competing Financial Interests statement**

The authors declare no competing financial interest.


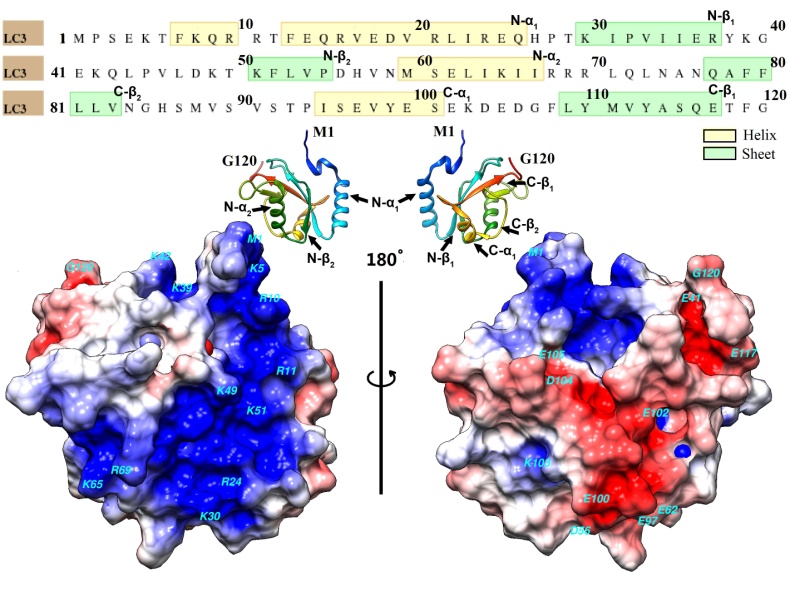


**Fig S1. Sequence (top) and electrostatic potential surface (bottom) of LC3-I protein.** The positive and negative charged part were colored by blue and red, respectively.


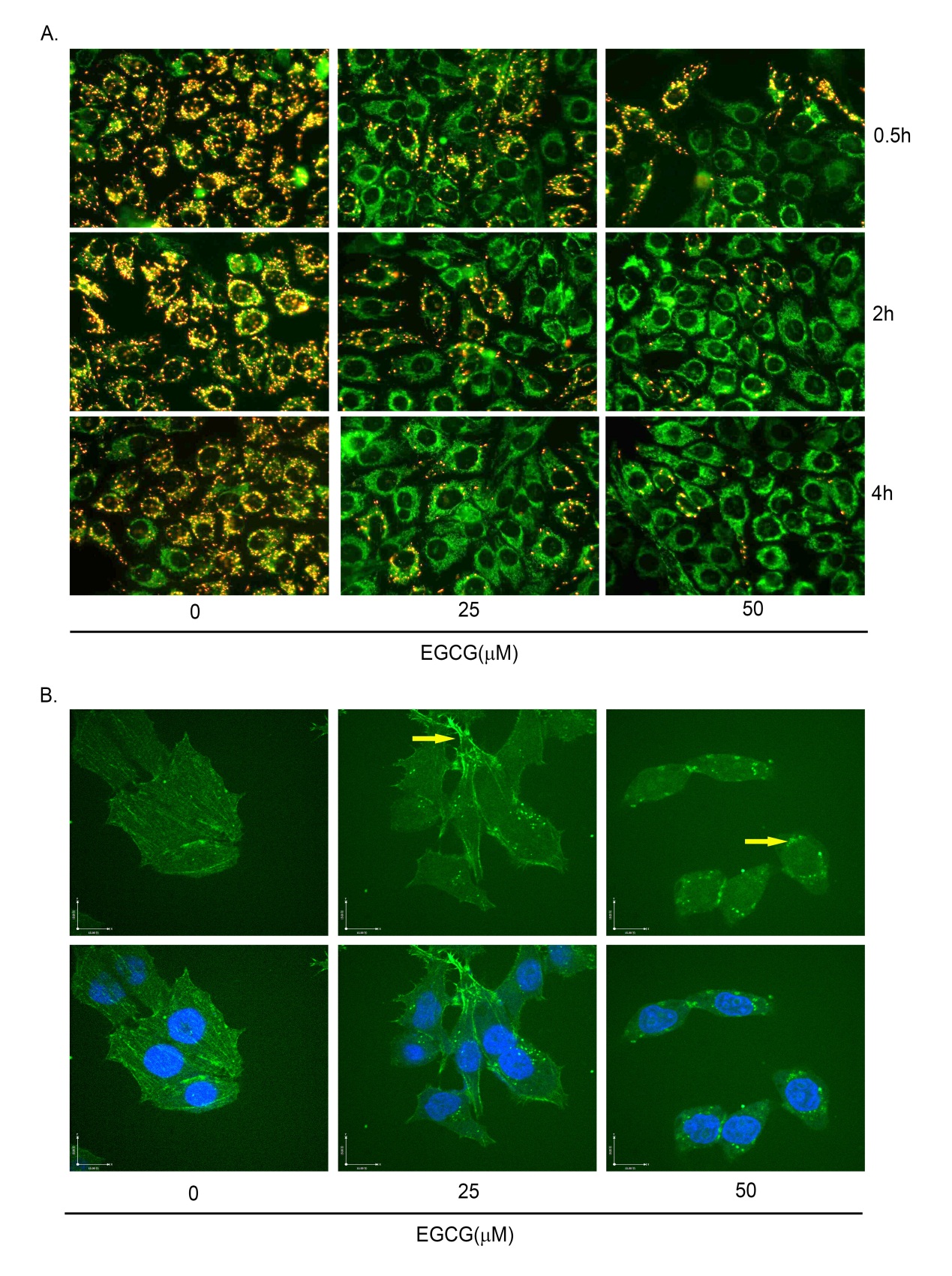
 **Fig S2. EGCG induces mitochondria and cytoskeleton damage in HepG_2_ cells.** (A) HepG_2_ cells were incubated with 0, 25, 50 μM EGCG for 0.5, 2, and 4 h and stained with JC-1 working buffer for 20min. The Images were captured using a fluorescence microscope (Cal Zeiss Microimaging).(×20)**.** (B) HepG_2_ cells were incubated with different concentration of EGCG(0, 25, 50 μM) for 6 h, and were fixed and stained with Actin-tracker Green(1:100)for 30min. The images detectin green actin were captured by a fluorescence microscope (Cal Zeiss Microimaging).(×60).


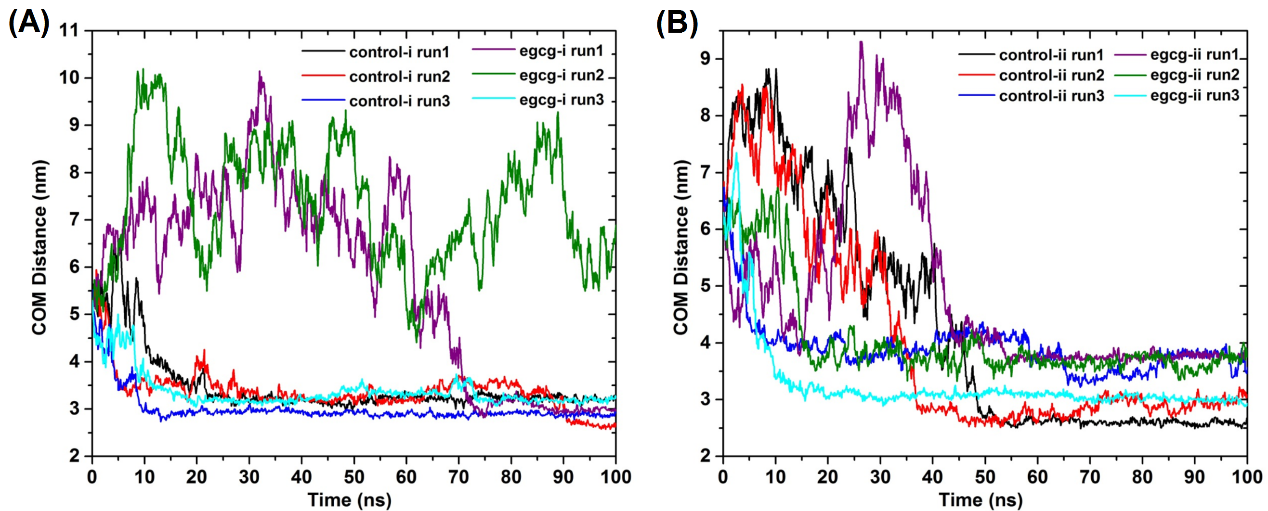


**Fig S3. EGCG induced inhibition of the dimerization of LC3-I.** The time evolution of center of Mass (COM) distance between two LC3-I proteins for all simulated trajectories of control-i and EGCG-i system (A), and control-ii and EGCG-ii system (B).


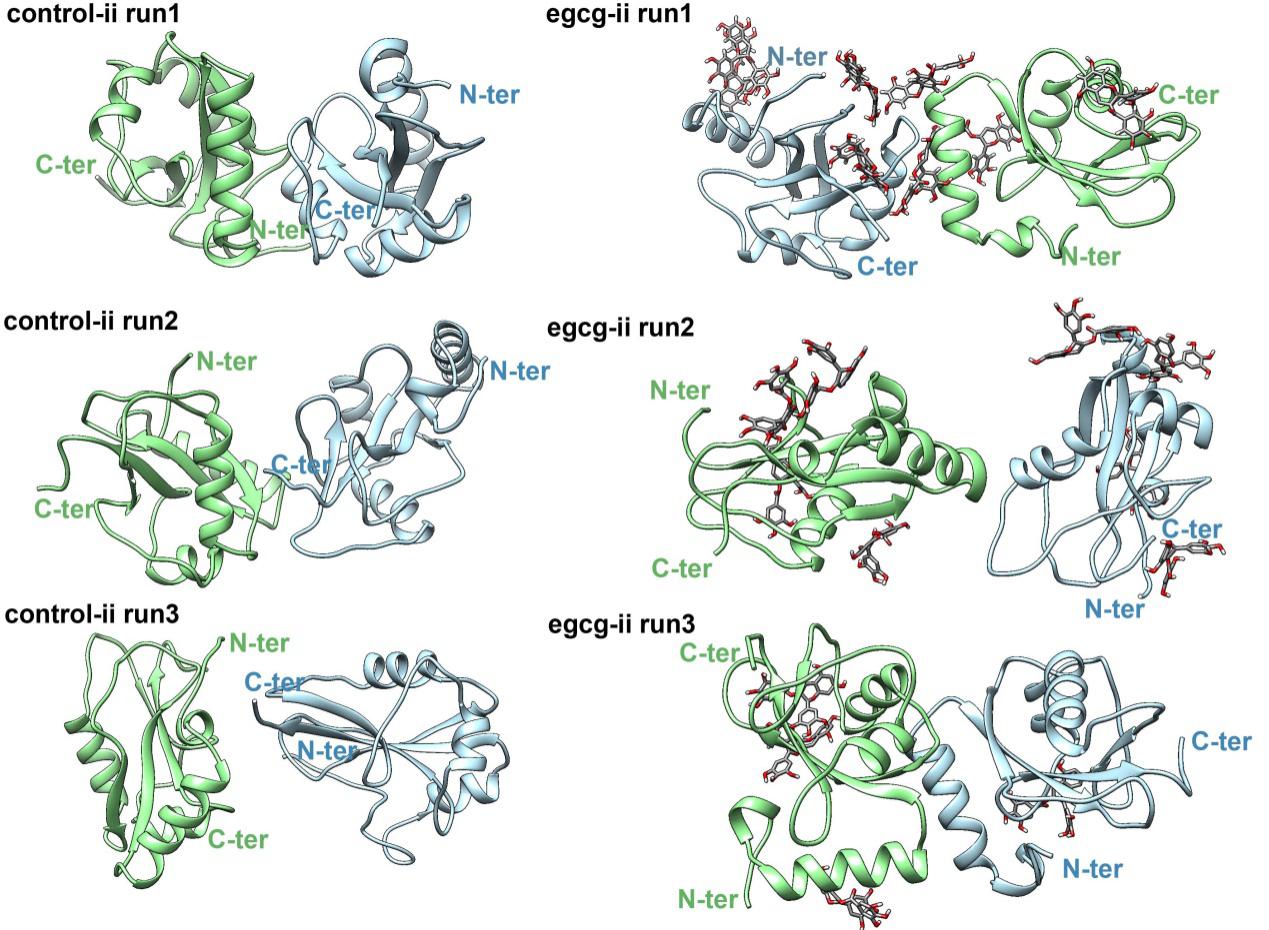


**Fig S4. The three final snapshots (at t = 100 ns) of control-ii (left column) and EGCG-ii system (right column, system depicted in Fig. 3) from different initial phase space.** LC3-I protein are displayed with different color to distinguish them, the terminal of proteins labeled N-ter and C-ter for same purpose.
